# Effects of a mobile-based self-management program on mental health in young adults: a randomized controlled trial

**DOI:** 10.3389/fpubh.2026.1830292

**Published:** 2026-06-17

**Authors:** Danbi Oh, Hye Seung Choi, David C. Mohr, Hyunjoo Na

**Affiliations:** 1 College of Nursing, The Catholic University of Korea, Seoul, Republic of Korea; 2 Department of Nutrition and Food Science, University of College Park, College Park, MD, United States; 3 Center for Behavioral Intervention Technologies (CBITs), Department of Preventive Medicine, Feinberg School of Medicine, Northwestern University, Chicago, IL, United States

**Keywords:** behavioral activation, mobile application, resilience, stress, young adults

## Abstract

**Background:**

Young adults in South Korea experience high stress from academic and economic pressures, but help-seeking and service use remain low. Mobile-based interventions have emerged as promising strategies to improve access to mental health support; however, empirical evidence for non-clinical young adult populations remains limited. The objective of this study was to evaluate the effectiveness of *DodaMe*, a mobile-based self-management program integrating positive psychology and behavioral activation, in improving mental health outcomes among community-dwelling young adults with elevated stress.

**Methods:**

This randomized controlled trial with a wait-list control group evaluated *DodaMe*, a 4-week mobile self-management program integrating Positive Psychology and Behavioral Activation, followed by a 4-week self-directed phase. Participants were recruited nationwide in South Korea from community-based youth and mental health service settings. A total of 179 young adults (aged 19–34) with elevated stress were randomized to the intervention (*n* = 92) or wait-list control group (*n* = 87). Stress (primary outcome) and depression, anxiety, and resilience (secondary outcomes) were assessed at baseline, 2, 4, and 8 weeks, and analyzed using generalized estimating equations. Week 8 program quality and app usage data were analyzed to assess acceptability and engagement.

**Results:**

The intervention showed a small, non-significant reduction in stress compared with the wait-list control group [B = −0.21, 95% *CI* (−0.41, 0.00)]. Resilience improved significantly, although the effect was small [B = 0.23, 95% *CI* (0.09, 0.37)]. No significant effects were found for depression or anxiety, with small effect sizes at 8 weeks. Participants evaluated the program positively, particularly its functionality and usefulness, while engagement data revealed frequent use of emotional check-ins and gratitude journaling.

**Conclusions:**

*DodaMe* did not significantly reduce stress but showed a small significant improvement in resilience among Korean young adults. The intervention may be a usable and potentially scalable approach for supporting mental health in community-dwelling young adults, but further refinement and adequately powered trials are needed to evaluate its effectiveness.

**Trial registration:**

ClinicalTrials.gov (NCT07174544).

## 1 Introduction

Mental health issues among young adults in South Korea have become increasingly prominent, often tied to structural problems such as employment competition and economic hardship. In recent years, South Korea has reported alarmingly high rates of depression and suicide among young adults, particularly those in their 20s and 30s (1, 2). According to statistics from the World Health Organization, South Korea consistently ranks among the countries with the highest global suicide rates, with young adults being significantly affected (1). The crisis stems from multiple factors, including intense academic and professional pressure, economic instability, and a highly competitive social environment (3). These factors create an environment in which stress and despair frequently diminish the importance of positive experiences and hinder the development of emotional wellbeing.

In response to this growing crisis, Korean mental health service demands of young adults have increased annually (1). Korean mental health welfare centers propose programs and initiatives specifically designed for young adults, with a significant number of these interventions delivered via mobile platforms. Among these, 12 mobile interventions have been developed featuring well-established and theory-based content (4). However, the high service demands of young adults and inconsistent quality of mental health services create a gap, making it difficult for providers to deliver standardized, evidence-based programs (5). To address this issue, it is necessary not only to validate the effectiveness of existing mental health mobile interventions but also to establish evidence that supports their application to individuals with diverse mental health symptoms.

Moreover, young Korean adults currently struggling with stress and mental health challenges require interventions that can provide positive stimulation and foster emotional resilience. One evidence-based approach is positive psychology interventions (PPIs), which have been shown to improve wellbeing and reduce psychological distress in young adults (6, 7). Positive psychology (PP), by emphasizing strengths and positive experiences, provides a practical framework for supporting young adults' personal growth and adaptive coping (8). Given the psychological burden young adults face, particularly from financial hardships, PP provides a relevant framework to address their mental health needs. However, Ciarrochi et al. (9) argued that conventional, complex PP intervention packages delivered in structured ways may not be sufficient to address individuals' varying and dynamic needs. They recommend a process-based approach in positive psychology—one that emphasizes personalized, contextually sensitive interventions, recognizes sociocultural and contextual variation, and provides a unified framework that bridges positive psychology with clinical psychology. This underscores the importance of developing accessible, targeted interventions rooted in PPIs to address the mental health needs of young adults.

In parallel with PPIs, another promising approach is Behavioral Activation (BA), a theoretically grounded intervention with a well-structured framework that supports the continuous reinforcement of healthy behaviors (10, 11). BA has demonstrated efficacy in mitigating psychological distress by encouraging engagement in healthy activities, which facilitate positive experiences, enhance motivation, and improve both thoughts and mood. Notably, its effectiveness has been substantiated across both traditional and digital platforms (11, 12). These therapeutic benefits of BA can be effectively extended through mobile platforms, making it a scalable option for young adult-centered interventions. A meta-analysis of BA-based mobile applications over the past decade demonstrated their effectiveness in alleviating depression and stress, and in enhancing quality of life (13). For young adults with mental health problems who avoid seeking help due to barriers such as stigma, limited access to care, or financial constraints, self-management programs delivered through mobile-based platforms have been recommended as a highly effective approach (14).

Therefore, this study aimed to evaluate a mobile self-management program that integrates Positive Psychology (PP) and Behavioral Activation (BA) as a mental health intervention for community-dwelling young adults with elevated stress in South Korea. Grounded in robust theoretical foundations, the program was designed to (1) facilitate experiences of positive affect, (2) support the discovery of personal character strengths, (3) promote strengths-based positive activities, and (4) enable planning for a positive and fulfilling life. Specifically, this randomized controlled trial examined the preliminary effects of the intervention on stress, depression, anxiety, and resilience, as well as its usability and perceived quality.

## 2 Methods

### 2.1 Study design

This study employed a randomized controlled trial with a wait-list control design to evaluate the effects of a mobile-based self-management program for young adults experiencing elevated stress in community settings in South Korea. The trial was conducted between September 2023 and December 2024. Participants were randomly assigned to either the intervention group or a wait-list control group. This randomized controlled trial was registered at ClinicalTrials.gov (NCT07174544).

### 2.2 Setting and participants

The target population consisted of community-dwelling young adults experiencing elevated levels of stress. Participants were recruited nationwide in South Korea through recruitment notices posted on offline and online bulletin boards of community-based organizations, including youth centers, community mental health and welfare centers, and a suicide prevention centers. The inclusion criteria were as follows: (i) aged 19–34 years at the time of participation, consistent with the definition of youth in the South Korean Framework Act on Youth (15), (ii) proficiency in using digital devices, (iii) use of an Android-based smartphone or tablet, (iv) a score of ≥14 on the Perceived Stress Scale-10 (PSS-10), indicating elevated perceived stress and consistent with thresholds used in prior mobile mental health intervention studies (16), and (v) provision of informed consent for random assignment.

**Figure 1:**
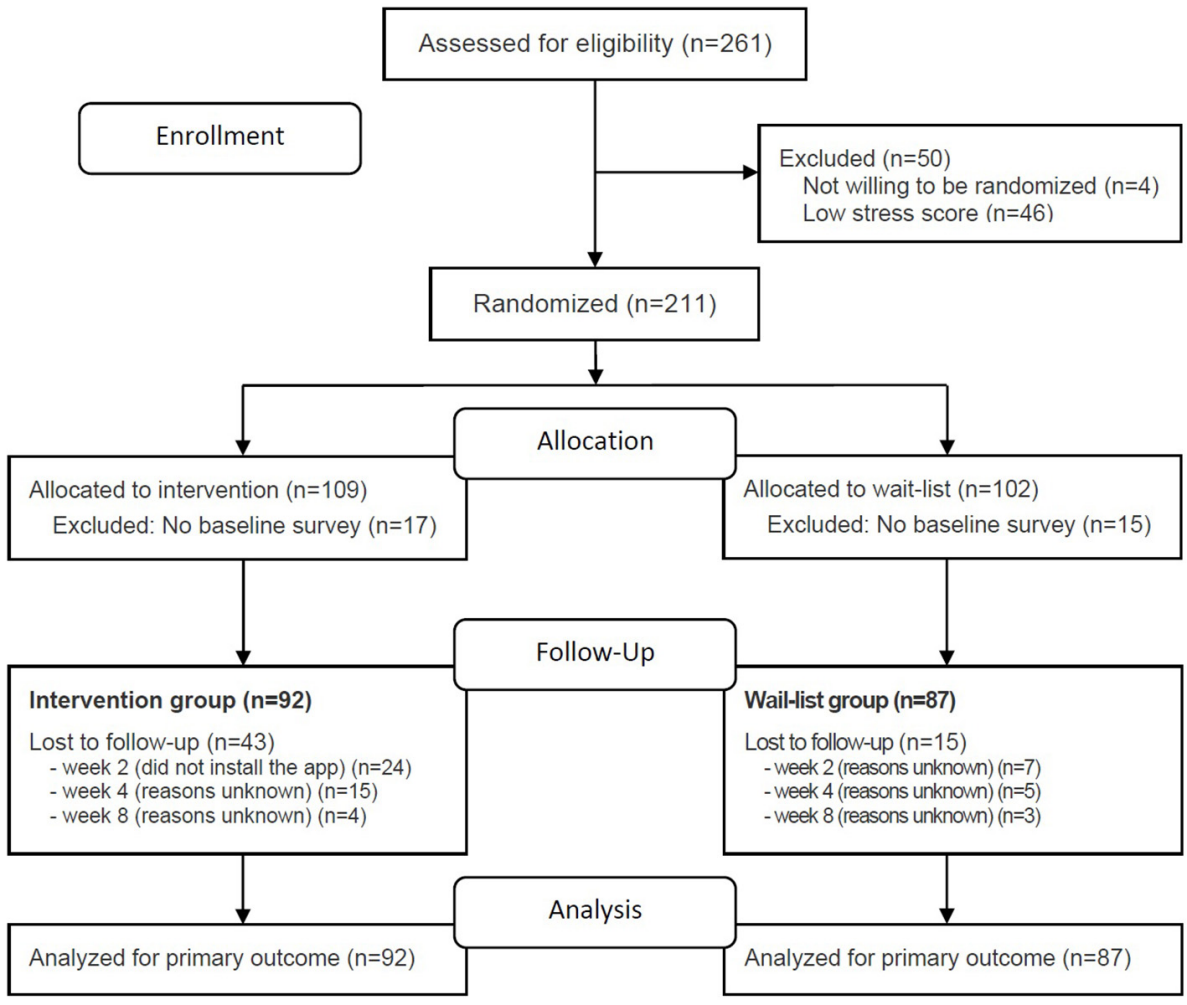
Study flow diagram.

The total of 261 individuals initially screened, 50 were excluded (46 participants scored below 14 on the PSS-10, and 4 declined to participate in the randomization process). A total of 211 participants provided informed consent and were assigned unique identification numbers. Using these de-identified IDs, participants were randomly allocated to either the intervention group or the wait-list control group through simple randomization with an online tool (https://www.randomizer.org/). Randomization was performed by a research assistant who was not involved in data collection or outcome assessment, and the principal investigator remained blinded to the allocation process to minimize allocation bias. Due to the nature of the mobile intervention and wait-list control design, participants and researchers could not be blinded to group assignment. However, outcome data were collected through self-report questionnaires, and data analysts were blinded to group allocation. Of the 211 participants randomized, 32 (17 intervention; 15 wait-list group) did not complete the baseline survey and therefore did not initiate participation in the trial, resulting in 92 participants in the intervention group and 87 in the wait-list group at study initiation. The flow of participants, including those retained for analysis, is presented in Figure 1.

### 2.3 Intervention

**Figure 2:**
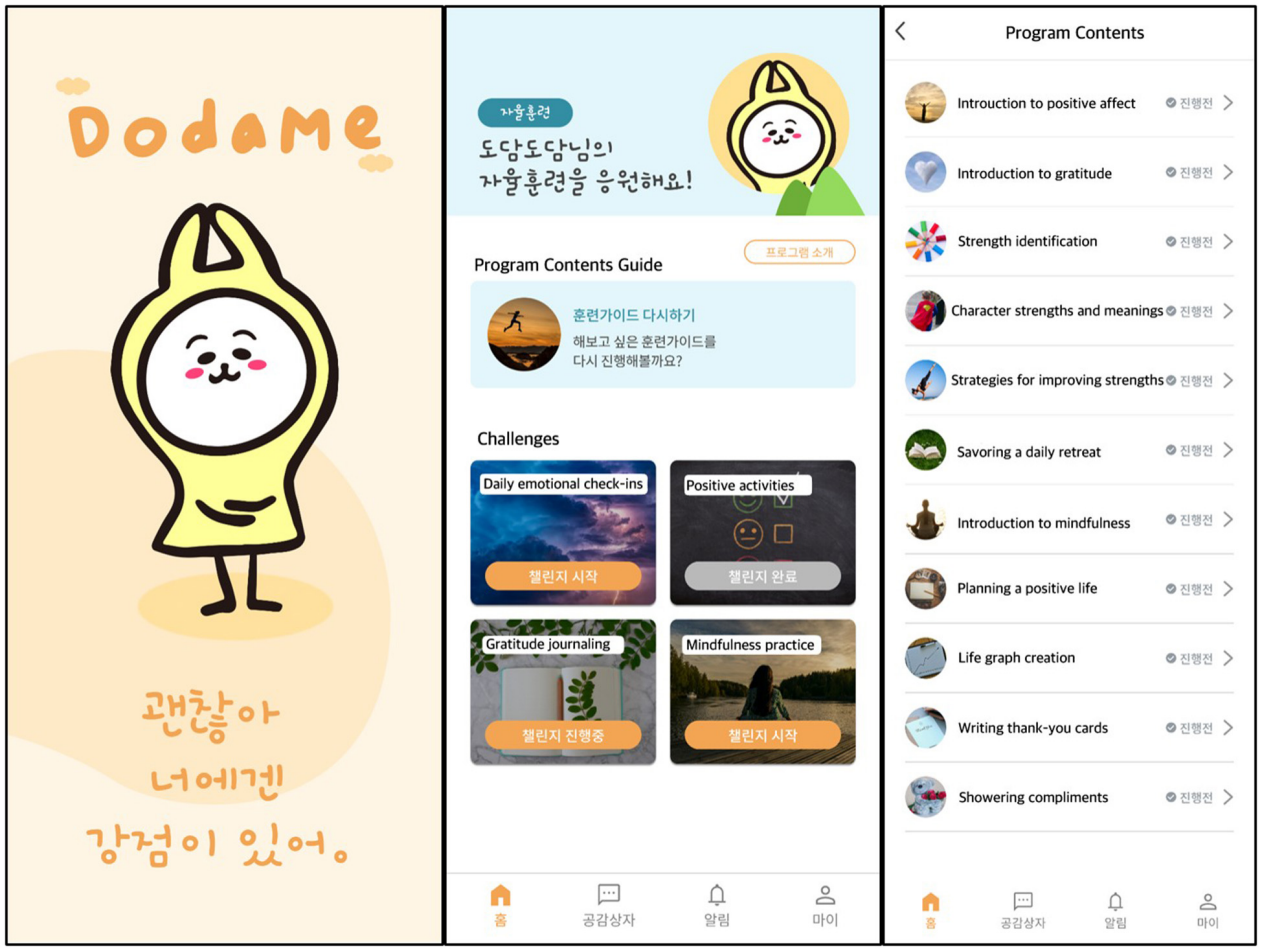
Key features and program components of *DodaMe*.

The intervention was delivered via an Android-based mobile application and was fully self-guided, with all content provided in a fixed, non-adaptive format. Participants accessed the program on their personal devices, and all intervention components were required. The program consisted of a 4-week guided intervention phase, followed by an additional 4-week self-directed period designed to encourage continued use (Table 1). The intervention program was structured to align with specific weekly objectives, incorporating psychoeducational PP contents and challenges to facilitate experiential learning and behavioral reinforcement using BA strategies. The weekly objectives were (1) experiencing positive affect, (2) discovering positive strengths, (3) utilizing positive strengths, and (4) planning a positive life. The challenges, including emotional check-ins, gratitude journaling, positive activity, and mindfulness practice, promoted engagement and reinforced positive behavioral changes by encouraging consistent participation. The key concepts of the mobile application are illustrated in Figure 2.

**Table 1:** Overview of the developed mobile self-management program.

Week	Objectives	Program contents (psychoeducation)	Challenges (action plan)	Engagement strategies
1	Experiencing positive affects	•Introduction to positive affect •Introduction to gratitude	•Emotional check-ins •Gratitude journaling	•Daily reminders • (default 8 p.m., customizable) •Activity planning and recording •Visual tracking • (Stamp map, • badge, calendar)
2	Discovering positive strengths	•Character strengths, assessment and feedback •Character strengths meaning •Strategies for improving strengths	•Strength identification •Strengths-based positive activities: based on highest-ranked strengths (10 per strength)	
3	Utilizing positive strengths	•Savoring a daily treat •Introduction to mindfulness	•Mindfulness practice •Strengths-based positive activities: based on highest-ranked strengths (10 per strength)	
4	Planning a positive life	•Planning a positive life •Life graph creation •Writing thank-you cards •Future goal setting	•Goal setting •Strengths-based positive activities: based on highest-ranked strengths (10 per strength)	
5–8	Strengthening Self-management strategies	•Showering compliments	•Self-selected positive activities •Engagement with all features	

The program content was tailored to each objective. Week 1 focused on the functions and benefits of positive affect and gratitude, delivered through challenges such as daily emotional check-ins and gratitude journaling. The daily emotional check-in allowed participants to monitor and record their overall mood using bipolar scales across multiple affective dimensions, including pleasant–unpleasant mood, depression–elevation, and anxiety–calmness. Gratitude journaling served as a gateway activity, in which participants recorded three things they were grateful for each day, reflected on these experiences, and could optionally upload related photos. Progression to the Week 2 intervention was contingent upon completing at least three days of gratitude journaling.

Week 2 focused on identifying and applying participants' character strengths based on the Values in Action (VIA) Classification of Character Strengths and Virtues (17). At the beginning of the week, participants completed a VIA assessment and received individualized feedback. The highest-ranked strength was automatically selected, and a set of recommended positive activities tailored to that strength was provided. A total of 10 activities were developed for each of the 24 character strengths. Participants were instructed to select three activities from the list, plan their implementation over the week, and complete all three as part of the weekly challenge. Completion of the activities was recorded within the app, and participants were encouraged to document their experiences.

Week 3 emphasized savoring and mindfulness practices to help participants learn, apply, and integrate their strengths into daily life. A total of 14 guided mindfulness exercises were developed and provided based on established guidelines to support stepwise stress reduction among young adults (18). In addition, positive activities targeting the second-highest VIA strength were implemented following the same procedures as in Week 2.

**Figure 3:**
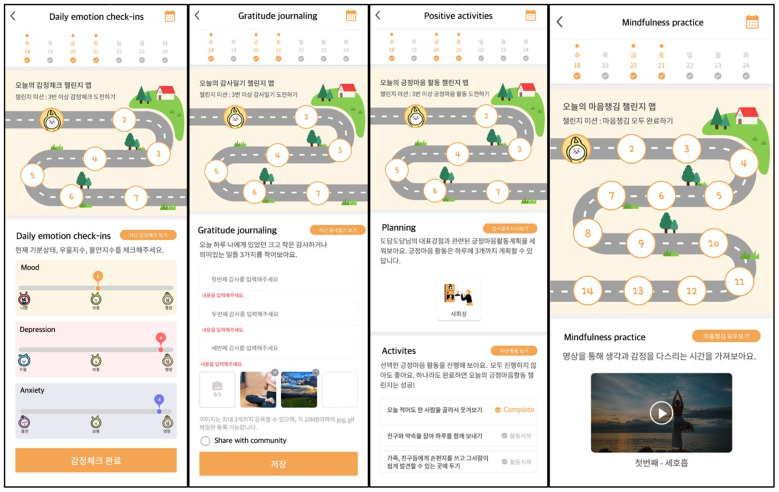
Action planning component of *DodaMe*.

Week 4 guided the participants in planning a positive future by creating a life graph, expressing gratitude through thank-you cards, and setting meaningful goals. Positive activities targeting the third-highest VIA strength were implemented following the same procedures as in Weeks 2 and 3. From Week 5 to Week 8, the program focused on enhancing self-management skills by encouraging participants to voluntarily select and engage in positive activities tailored to their identified character strengths. Each participant received a set of 10 recommended activities aligned with their strengths, allowing them to practice self-directed behavioral changes (Figures 2, 3).

**Figure 4:**
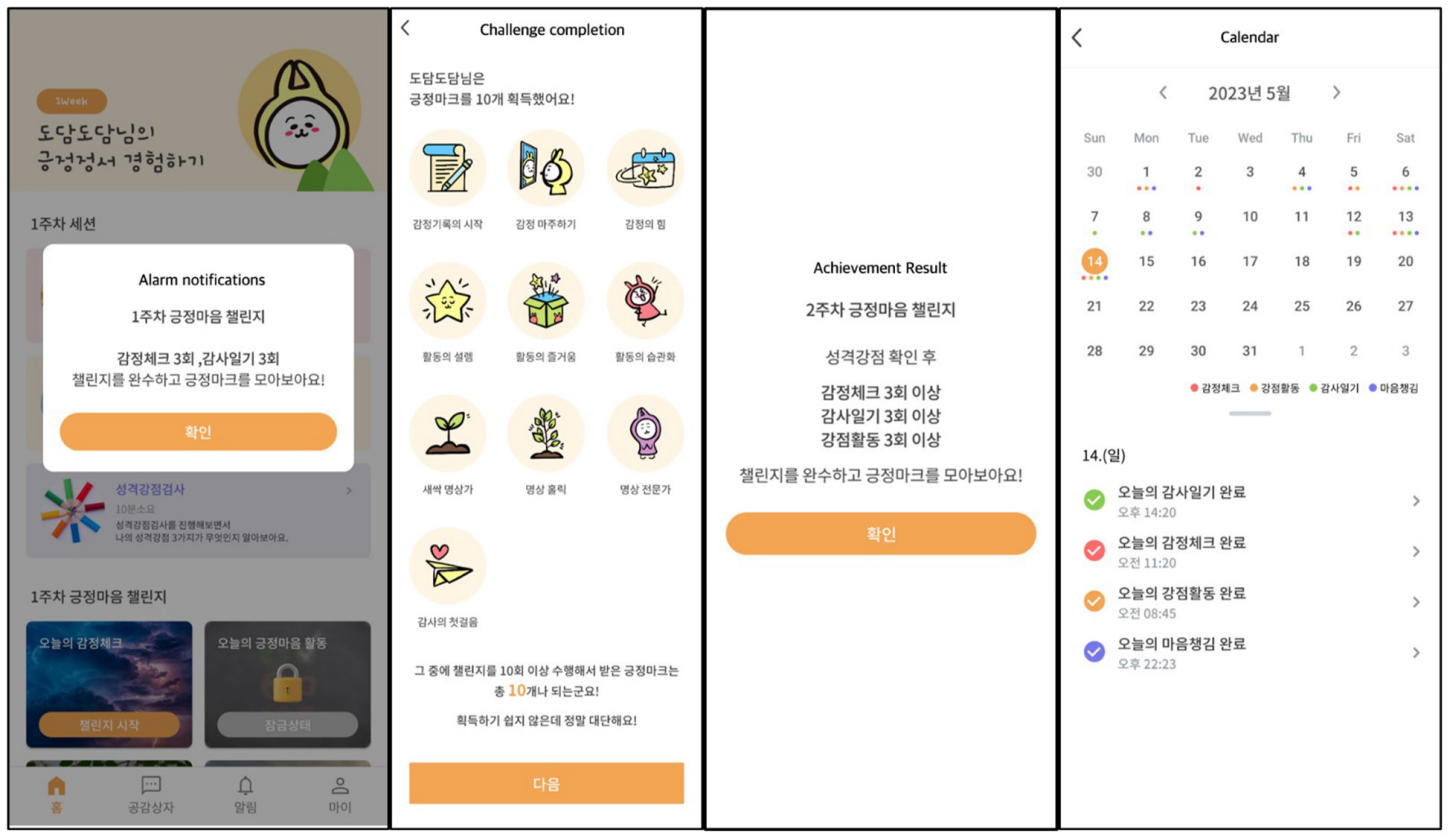
Engagement strategies used in *DodaMe*.

Participants were able to engage with the program up to five times per week, with each session expected to take approximately 5–10 min. Daily reminders were scheduled at 8:00 p.m. by default; however, participants were prompted during initial setup to select a preferred notification time. Adherence was defined differently across weeks. In Week 1, adherence was determined by completing at least three days of gratitude journaling. In Weeks 2–4, adherence was defined as planning and completing the prescribed strength-based positive activities and recording their completion within the app. Engagement was supported through automated reminders, visual progress tracking, and feedback mechanisms within the app. Completion of activities was recorded automatically, and participants received reinforcement through reward badges and encouragement messages based on their activity completion. Progress was visually represented using a stamp-based tracking map and a calendar displaying the frequency of completed entries (Figure 4).

The program objectives, contents, challenges, and engagement strategies were validated by experts in psychology and psychiatric nursing. The mobile functions were reviewed by information technology specialists to ensure appropriate algorithm-based delivery. The application's design emphasized user experience and usability, incorporating feedback from prospective users, while visual elements such as icons and illustrations were created in collaboration with young artists to align with young adults' preferences.

### 2.4 Measurements

At baseline, participants completed a demographic survey along with initial assessments of the primary outcome (stress) and secondary outcomes (depression, anxiety, and resilience). Follow-up assessments of these psychological measures were conducted at weeks 2, 4, and 8 in both the intervention and wait-list control groups. In addition, at week 8, participants in the intervention group completed a survey evaluating the perceived quality of the mobile program. Engagement with the program was further examined using app-recorded usage data collected throughout the intervention period.

*Demographics* were assessed using three questions regarding age, sex and residential area.

*Stress* was the primary outcome and measured using the Korean version of Perceived Stress scale (PSS-10) (19, 20), which consists of ten items rated on a 5-point Likert scale. The total score ranges from 0 to 40, with higher scores reflecting greater perceived stress. Although a specific cut-off point was not provided for this scale, previous studies were referenced, and a score of 14 was used to define moderate-to-high levels in this study (16). The internal consistency of the scale in this study was α = 0.70.

*Depression* was assessed using the Korean version of the Patient Health Questionnaire-9 (PHQ-9) (21), which includes nine items evaluating depressive symptoms. Each item is rated on a 4-point Likert scale, indicating the frequency of symptoms over the past two weeks. The total score ranges from 0 to 27, with higher scores representing more severe depression. In this study, the Cronbach's alpha for the scale was 0.84.

*Anxiety* was assessed using the Generalized Anxiety Disorder Scale (GAD-7) (22), which includes seven items that measure anxiety symptoms. Each item is rated on a 4-point Likert scale, reflecting the frequency of symptoms over the last two weeks. The total score ranges from 0 to 21, with higher scores indicating more severe anxiety. The internal consistency in this study was α =0.88.

*Resilience* was measured using the Korean version of the Brief Resilience Scale (BRS) (23, 24), which consists of six items rated on a 5-point Likert scale. The mean score was calculated, with higher scores indicating greater resilience. In this study, the internal consistency of the scale was α =0.71.

*Quality of the mobile program* was assessed using the Korean version of the end-user Mobile Application Rating Scale (uMARS) (25, 26), which evaluates the quality of mobile health apps. It consists of 20 items divided into five subscales–engagement, functionality, aesthetics, information quality, and subjective quality–along with an additional 6-item perceived impact of mobile program subscale. The items were evaluated on a five-point Likert scale (1 = Inadequate to 5 = Excellent). The internal consistency of the uMARS in this study was 0.95.

*Engagement* with the developed mobile program was assessed by measuring the total usage frequency of its content, including four daily challenges: emotional check-ins, positive activity, gratitude journaling, and mindfulness practice. The participants' engagement levels were determined based on how frequently they recorded their activity usage for each challenge.

### 2.5 Study procedure

Prospective participants were recruited through offline and online platforms using posters containing a QR code that linked to a study information webpage. The posters described the study's objectives, methodology, potential benefits, data confidentiality, voluntary participation, and possible risks. Individuals who completed the online screening questionnaire and provided contact information received an informed consent form via email. Those who returned signed consent forms and met the eligibility criteria were enrolled.

Participants assigned to the intervention group were sent a text message with a link to install the *DodaMe* app. The intervention lasted eight weeks, consisting of a structured four-week phase with weekly objectives, followed by a self-directed phase (weeks 5–8) in which participants voluntarily practiced selected strategies. Assessments were completed in-app at baseline, weeks 2, 4, and 8.

Participants in the wait-list control group did not receive any intervention during the 8-week study period. They were asked to complete the same assessments at identical time points (weeks 0, 2, 4, and 8) via survey links delivered by text message. After week 9, participants in the wait-list group were granted access to the *DodaMe* app.

*DodaMe* was developed by the research team in collaboration with app developers during 2023–2024. The app was available on the Google Play Store from September 2023 to December 2024 but is not currently publicly accessible, as further technical and content refinement is required before broader dissemination.

### 2.6 Data analysis

A priori power analysis using G^*^Power (version 3.1) for repeated-measures ANOVA indicated that 128 participants (64 per group) were required to detect a medium effect size (*f* = 0.25) with 80% power at a two-tailed alpha of 0.05. The sample size calculation was based on an expected medium effect size, informed by prior evidence of a smartphone-based stress intervention conducted among workers (27). Allowing an anticipated dropout rate of 40% (28), the target sample was set at approximately 180. To ensure adequate power, 211 participants were enrolled, and 92 intervention and 87 wait-list participants were included in the final analysis. The final analytic sample exceeded the minimum requirement, indicating sufficient statistical power to detect meaningful group differences over time. Stress was designated as the primary confirmatory endpoint, while depression, anxiety, and resilience were treated as secondary exploratory outcomes.

Data were analyzed using IBM SPSS version 27 (29). Normality of the data was evaluated using the Kolmogorov-Smirnov Test. Descriptive statistics were used to summarize the demographic characteristics of study participants, as well as program quality and engagement. Group homogeneity at baseline and for the measured variables was tested using the independent *t*-tests and chi-squared test. A conservative intention to treat (ITT) approach was applied to maintain the integrity of group assignments and outcomes, preserving the original balance from random assignment. A modified intention-to-treat approach was used, including all randomized participants who completed the baseline assessment, with missing follow-up data addressed using multiple imputation. Missing follow-up data were imputed using multivariate imputation by chained equations (MICE) and 20 imputed datasets. The imputation model included all outcome variables (stress, depression, anxiety, and resilience) across all time points as well as demographic variables (age, sex, and residential area), assuming data were missing at random (MAR). The imputed datasets were subsequently analyzed using generalized estimating equations (GEE). GEE models were specified using a normal distribution with an identity link function, an exchangeable working correlation structure, and robust sandwich variance estimators. Group was coded as a binary variable. Time was modeled as a continuous variable representing weeks, and the group-by-time interaction term was specified to test linear differences in outcome trajectories between groups over time. Baseline measurements were included as repeated observations in the longitudinal models and were not entered as separate covariates. Effect sizes (Cohen's *d*) were computed at the 8-week assessment to quantify the magnitude of between-group differences. Sensitivity analyses were performed using a completer approach, including only participants with complete data at all assessment points, applying the same GEE modeling framework as in the primary analyses.

### 2.7 Ethical statement

Ethical approval for the study was granted from the university's Institutional Review Board (IRB No. MC23FNSI0059). Prior to participation, all individuals provided written informed consent after receiving a full explanation of the study and having the opportunity to ask questions. Participant confidentiality was strictly maintained, and individuals were informed that they could decline or withdraw from the study at any time without adverse consequences. No harms or unintended events were reported in either the intervention or wait-list control group.

## 3 Results

### 3.1 Baseline characteristics of demographic and measured variables

Figure 1 displays the CONSORT Diagram. Among the 179 participants, 92 were assigned to the intervention group and 87 to the wait-list group. Attrition rates differed substantially between groups. In the intervention group, dropout rates were 26.1% at week 2 (*n* = 24), 42.4% at week 4 (*n* = 39), and 46.7% at week 8 (*n* = 43). All participants lost to follow-up at week 2 had not installed the application. At later time points, specific reasons for dropout were not systematically collected. Baseline characteristics of the study participants are presented in Table 2. The mean age was 27.85 years (SD = 4.16) in the intervention group and 27.46 years (SD = 4.42) in the wait-list group. Females comprised the majority in both groups, with 79 (85.87%) in the intervention group and 72 (82.76%) in the wait-list group. Regarding residential area, 57 (61.96%) participants in the intervention group and 50 (57.47%) in the wait-list group resided in metropolitan areas, while 35 (38.04%) and 37 (42.53%) participants, respectively, were from small or medium cities. There were no significant differences between groups in demographic characteristics. The groups were comparable on all baseline variables except for stress and resilience. Specifically, the intervention group showed higher baseline stress scores (M = 22.64 vs. 19.76, *p* < 0.001) and lower resilience scores (M = 15.24 vs. 17.79, *p* < 0.001) compared to the wait-list group.

**Table 2:** Baseline demographic characteristics and the measured variables.

Characteristics	Intervention group (*n* = 92)	Wait-list group (*n* = 87)	t or χ2	*p*
	*N* (%) or M ±SD	*N* (%) or M ±SD		
				(*N* = 179)
Age (years)	27.85 ± 4.16	27.46 ± 4.42	0.605	0.546
Sex			0.328	0.567
Female	79(85.87)	72(82.76)		
Male	13(14.13)	15(17.24)		
Residential area			0.374	0.541
Metropolitan	57(61.96)	50 (57.47)		
Small and medium city	35 (38.04)	37(42.53)		
Stress	22.64 ± 5.82	19.76 ± 3.64	3.996	<0.001
Depression	10.21 ± 5.89	9.24 ± 5.42	1.140	0.256
Anxiety	8.20 ± 5.75	7.68 ± 4.88	0.647	0.518
Resilience	15.24 ± 5.06	17.79 ± 1.71	−4.575	<0.001

M, Mean; SD, Standard Deviation.

### 3.2 Group differences over time in study variables

Table 3 presents a comparison of descriptive variables between groups over time. Table 4 presents the results of GEE analysis examining group differences and time effects over 8 weeks on the primary outcome (stress) and secondary outcomes (depression, anxiety, and resilience), along with effect sizes (Cohen's d) at the 8-week assessment.

**Table 3:** Descriptive variables between groups at baseline, 2, 4, and 8 weeks.

Variable	Week	Intervention group		Wait-list group	
		*n*	M ±SD	*n*	M ±SD
					(*N* = 179)
Stress	0	92	22.64 ± 5.82	87	19.76 ± 3.64
	2	68	20.57 ± 5.78	80	19.00 ± 3.98
	4	53	20.08 ± 6.62	75	18.71 ± 4.00
	8	49	19.41 ± 6.20	72	18.35 ± 4.05
Depression	0	92	10.21 ± 5.89	87	9.24 ± 5.42
	2	68	8.34 ± 5.44	80	8.51 ± 5.63
	4	53	7.72 ± 5.91	75	7.95 ± 5.36
	8	49	8.37 ± 6.38	72	7.69 ± 5.50
Anxiety	0	92	8.20 ± 5.75	87	7.68 ± 4.88
	2	68	6.88 ± 5.65	80	7.13 ± 5.23
	4	53	6.43 ± 5.61	75	7.03 ± 5.21
	8	49	7.63 ± 6.11	72	6.36 ± 4.87
Resilience	0	92	15.24 ± 5.06	87	17.79 ± 1.71
	2	68	15.37 ± 5.10	80	17.59 ± 1.76
	4	53	16.06 ± 5.68	75	17.87 ± 1.93
	8	49	16.14 ± 5.87	72	17.54 ± 1.85

M, mean; SD, standard deviation.

**Table 4:** Generalized estimating equations analysis of group differences and time effects on variables.

Variable	B	SE	95% *CI*	Wald χ^2^	*p*	Cohen's *d* (8week)
						(*N* = 179)
Stress						0.23
Group	2.56	0.66	1.26, 3.86	14.96	0.000	
Time	−0.16	0.06	−0.28, −0.05	7.70	0.006	
Group x time	−0.21	0.11	−0.41, 0.00	3.83	0.050	
Depression						0.19
Group	0.72	0.81	−0.86, 2.91	0.80	0.371	
Time	−0.20	0.07	−0.34, −0.05	6.92	0.009	
Group x time	0.01	0.13	−0.24, 0.25	0.01	0.942	
Anxiety						0.24
Group	0.28	0.75	−1.19, 1.76	0.14	0.706	
Time	−0.18	0.06	−0.30, −0.05	7.87	0.005	
Group x time	0.08	0.11	−0.13, 0.29	0.53	0.465	
Resilience						0.19
Group	−2.53	0.51	−3.53, −1.52	24.34	0.000	
Time	−0.04	0.04	−0.11, 0.03	−1.18	0.239	
Group x time	0.23	0.07	0.09, 0.37	10.67	0.001	

SE, standard error; CI, confidence interval. Effect sizes (Cohen's d) represent between-group differences at the 8-week assessment and are not derived from GEE coefficients.

The primary outcome, stress, showed a non-significant group-by-time interaction [B = −0.21, 95% *CI* (−0.41, 0.00), *p* = 0.050]. The effect size at 8 weeks was small (*d* = 0.23). Resilience showed a significant group-by-time interaction [B = 0.23, 95% CI (0.09, 0.37), *p* = 0.001], indicating a greater increase over time in the intervention group than in the wait-list control group, although the 8-week effect size was small (*d* = 0.19). No significant group-by-time interactions were found for depression or anxiety, and the 8-week effect sizes were small (*d* = 0.19 and 0.24, respectively) (30).

As a sensitivity analysis, a completer analysis was conducted including only participants with complete data at all assessment points (*n* = 121; intervention: *n* = 49, wait-list: *n* = 72). For stress (B = −0.24, *p* = 0.059) and resilience (B = 0.23, *p* = 0.005), the group-by-time interaction effects were attenuated. The interaction effect for stress was not statistically significant, whereas the effect for resilience remained significant. The results for depression (B = 0.02, *p* = 0.890) and anxiety (B = 0.07, *p* = 0.498) were consistent with the primary analyses.

### 3.3 Quality and engagement of the program

Among the 49 participants in the intervention group who completed the 8-week program, 44 participants completed the app engagement and quality evaluation. The mean score for application quality, which included the domains of engagement, functionality, aesthetics, and information, was 3.82 (SD = 0.66). Functionality received the highest rating with a mean score of 4.14 (SD = 0.71). The mean score for subjective quality was 3.09 (SD = 0.79). The perceived impact of the mobile program had a mean score of 3.80 (SD = 0.80). Engagement with the developed program was measured based on the usage frequency of the mobile program. The median frequency of use of the app's features was 28 for emotional check-ins, 25 for gratitude journaling, 14 for positive activities, and 14.5 for mindfulness practice during the 8 weeks (Table 5). When engagement data were examined for all 92 intervention participants, median usage frequencies were lower across all features: emotional check-ins (Mdn = 21.50), gratitude journaling (Mdn = 15.50), positive activities (Mdn = 10.00), and mindfulness (Mdn = 8.50).

**Table 5:** Quality and engagement of the developed mobile program.

Variables	Categories	Mean (SD)	Median	IQR [Q1–Q3]
				(*N* = 44)
Quality	App quality	3.82 (0.66)		
	Engagement	3.28 (0.65)		
	Functionality	4.14 (0.71)		
	Aesthetics	3.83 (0.81)		
	Information	4.02 (0.80)		
	Subjective quality	3.09 (0.79)		
	Perceived impact	3.80 (0.80)		
Usage frequency	Emotional check-in	37.59 (28.02)	28.00	11.75 [24.25–36.00]
	Gratitude journaling	31.16 (22.77)	25.00	11.50 [20.25–31.75]
	Positive activity	20.68 (20.29)	14.00	10.00 [11.00–21.00]
	Mindfulness	18.70 (18.75)	14.50	10.25 [9.25–19.50]

SD, Standard Deviation; IQR, Interquartile Range.

## 4 Discussion

This study aimed to address the growing need for effective stress management strategies for community-dwelling young adults in South Korea by developing and implementing *DodaMe*, an integrative mobile-based self-management intervention grounded in positive psychology and behavioral activation. The intervention is theoretically well grounded and systematically structured for effective intervention delivery. It employs an algorithm-driven approach to weekly activity planning, tracking, positive reinforcement, and reminders to ensure a structured, step-by-step engagement process.

The effects of this intervention were evaluated among young adults experiencing moderate to high stress, with outcomes measured relative to a wait-list control condition. A nationwide sample of 179 participants, comprising 92 in the intervention group and 87 in the wait-list control group, was included in this study. Participants had an average age of 27 years, with over 80% identifying as female and the majority living in metropolitan areas. The primary outcome was perceived stress, while secondary outcomes included depression, anxiety, and resilience. All outcomes were assessed at baseline, 2 weeks, 4 weeks, and at the end of the self-directed practice period (8 weeks). Baseline stress and resilience differed between groups, with the intervention group showing higher stress and lower resilience at baseline. These imbalances may reflect chance variation associated with simple randomization in moderate sample sizes and should be considered when interpreting changes in outcomes over time (31).

The primary outcome, stress, decreased over time; however, the group-by-time interaction was not statistically significant, indicating no significant effect of the mobile self-management program. This finding aligns with previous meta-analyses demonstrating that mobile-based mental health interventions incorporating positive psychology and behavioral activation yield beneficial effects on stress (32, 33). Participants in this study frequently used core app features—particularly emotional check-ins and gratitude journaling—and most engaged at least once with mindfulness components. This pattern reflects the mechanisms highlighted by Weisel et al. (34), emphasizing the importance of real-time self-monitoring and repeated use of app-based interventions for reducing stress. Moreover, repeated monitoring and mindfulness practice may have enhanced users' self-awareness and self-acceptance, thereby reducing reactivity to negative emotions and stressors. This interpretation is consistent with prior evidence from a mindfulness meditation mobile app trial showing improvements in stress-related outcomes among college students with elevated stress (16). Although the initial sample size met a priori power requirement, the calculation was based on an expected medium effect size informed by prior smartphone-based stress intervention research (27), whereas the observed effect on stress was small and non-significant. This may have reduced the effective sample size and limited the study's ability to detect small intervention effects.

Among the secondary outcomes, resilience scores in the intervention group showed a significant improvement compared to the wait-list group, suggesting that the mobile self-management intervention may support psychological resilience in young adults. The results of resilience interventions demonstrate small-to-moderate effects on enhancing coping, emotional wellbeing, and reducing psychological symptoms (35). This improvement in resilience likely contributes to improved stress coping and mental health outcomes, supporting the utility of app-based mental health programs in preparing individuals to manage future challenges effectively (36). These findings can also be interpreted through the lens of the Broaden-and-Build Theory (37), which posits that positive emotions broaden individuals' cognitive and behavioral repertoire and, over time, build enduring personal resources such as resilience. In our program, gratitude journaling encouraged participants to repeatedly recognize and savor positive emotions, while positive activity prompts provided opportunities to practice adaptive coping strategies in daily life. Together, these elements may have broadened participants' perspectives and gradually built psychological resources, thereby strengthening their resilience and capacity to manage stress more effectively. Furthermore, resilience building is particularly critical during young adulthood, a developmental stage characterized by career uncertainty, identity formation, and increasing independence (38). Strengthening resilience during this formative period may help buffer the negative impact of stress and promote adaptive coping and long-term psychological wellbeing. Because resilience was specified as an exploratory outcome rather than a confirmatory endpoint in this study, the observed improvement should be replicated in future confirmatory studies.

There were no significant group-by-time differences in depression and anxiety. Previous research has generally found that mobile-based interventions, including mindfulness programs, are effective in reducing depressive and anxiety symptoms, with meta-analytic evidence indicating small but significant effects on depression and anxiety (33, 39). However, those studies typically involved participants with clinically elevated symptoms of depression or anxiety, whereas the current study targeted young adults experiencing heightened stress but not necessarily clinical psychological symptoms, which may explain the absence of group differences. In addition, our program emphasized activities that fostered positive emotions and recommended behaviors to cope with stress, which may have been less directly impactful on clinical psychiatric symptoms but more effective in enhancing resilience and stress management.

Among participants who completed the intervention, app quality was rated positively, particularly for functionality, and the program was perceived as having a meaningful impact. Engagement data indicated frequent use of key features, with emotional check-ins and gratitude journaling used most often. Participants rated overall quality positively, with scores exceeding 3 out of 5 on average. Previous studies using uMARS have reported similar findings, with most mobile mental health apps receiving above-average scores across total and subscale ratings (40, 41), which are consistent with the present results. Participants were encouraged to complete emotional check-ins, gratitude journaling, and positive activities at least three times per week, with 14 mindfulness exercises available throughout the program. The participation in the challenges was generally in line with the recommended usage, indicating consistent adherence to the intervention guidelines. Additionally, the mindfulness usage data suggests that participants who engaged with the app experienced all scenarios at least once. These findings suggest that the intervention was acceptable among completers; however, they should be interpreted with caution, as quality of app and engagement data were available for only 44 of the 92 participants in the intervention group. Variations in usage among completers suggest that some intervention components were more engaging than others; however, these patterns may have been influenced by selective retention of more engaged participants. Future studies should explore more flexible and user-directed intervention structures that allow participants to select preferred activities or intervention components and evaluate their differential effects on engagement and outcomes.

Although the intervention demonstrated potential benefits in enhancing resilience among young adults with moderate stress, several limitations should be acknowledged. First, the generalizability of the findings is limited by the predominantly female sample and the restriction to Android users. Future studies should include more diverse populations and broader platform accessibility. Second, baseline differences in stress and resilience between groups were observed despite randomization. Third, the internal consistency of the stress measure was acceptable (Cronbach's α = 0.70) but at the lower bound, which may have reduced measurement sensitivity for detecting intervention-related changes in the primary outcome. Fourth, the high dropout rate in the intervention group may have reduced the effective sample size and introduced completer bias. Although attrition is common in smartphone-based trials (42), the completer-based sensitivity analysis showed generally similar patterns to the primary analysis. Finally, the wait-list control condition did not account for non-specific factors such as attention, expectancy, or Hawthorne effects, which may have influenced the observed outcomes. Prior evidence suggests that wait-list controlled trials may overestimate intervention effects compared with usual-care control conditions (43). Therefore, the intervention effects observed in this study should be interpreted cautiously.

Despite these methodological limitations, this study highlights the potential of evidence-based mobile interventions incorporating positive psychology and behavioral activation approaches into community mental health practice. Future studies should consider more adaptive and user-directed intervention frameworks, including component-based or factorial designs, to enhance engagement, reduce attrition, and identify the most effective intervention elements for individual users. From an implementation perspective, mental health professionals could leverage such tools to complement existing services, particularly for young adults who may be reluctant to seek formal treatment due to stigma, accessibility, or cost barriers. The program may also be adopted in university counseling centers to support students who do not have clinical diagnoses but wish to monitor and maintain their mental health. Furthermore, its emphasis on positive experiences and resilience aligns well with preventive mental health approaches. To enhance its practical application, mental health services could consider incorporating *DodaMe* as a supplementary intervention in youth counseling programs, educational settings, and community mental health initiatives.

*DodaMe* did not significantly reduce the primary outcome, stress, but showed a small significant improvement in resilience as a secondary outcome. The program's usability and perceived quality were rated positively, and user engagement was consistent with recommended guidelines. Taken together, these findings suggest that *DodaMe* may be a usable, engaging, and potentially scalable approach for supporting mental health in non-clinical community settings. Further refinement and adequately powered trials with more rigorous control conditions are needed to evaluate its effectiveness.

## Data Availability

The raw data supporting the conclusions of this article will be made available by the authors, without undue reservation.
